# Influence of pre- and post-usage flushing frequencies on bacterial water quality of non-touch water fittings

**DOI:** 10.1186/1471-2334-13-402

**Published:** 2013-08-30

**Authors:** Miranda Suchomel, Magda Diab-Elschahawi, Michael Kundi, Ojan Assadian

**Affiliations:** 1 Institute of Hygiene and Applied Immunology, Medical University of Vienna, Kinderspitalgasse 15, Vienna 1090, Austria; 2 Clinical Institute for Hospital Hygiene, Medical University of Vienna, Waehringer Guertel 18-20, Vienna 1090, Austria; 3 Institute of Environmental Health, Medical University of Vienna, Kinderspitalgasse 15, Vienna 1090, Austria

## Abstract

**Background:**

Non-touch fittings have been reported to be susceptible for *Pseudomonas aeruginosa* accumulation. A number of factors may contribute to this, including the frequency of usage, duration of water stagnation, or presence of plastic materials. Programmable non-touch fittings are appearing which allow regular automated post-flushing with cold water to prevent water stagnation. However, the ideal duration of post-flushing is unknown as well as the effect of pre-rinsing with cold water before use.

**Methods:**

Eight non-touch fittings with brass valve blocks were mounted on a mobile test sink and connected to the same central water pipe source, differing only in presence or absence of water connection pipes, length of connection pipe, frequency of usage, and time intervals for pre- and post-usage water flush. The total bacteria colony-forming unit (cfu) counts were obtained by the spread plate technique.

**Results:**

Low frequency of water use in combination with a long stagnating water column resulted in high bacterial cfu counts. Post-usage flushing for 2 seconds did not differ from no flushing. Flushing for 10 seconds with cold water after use or 30 seconds flush before use were both the most effective measures to prevent non-touch fittings from biofilm formation over a period of 20 weeks.

**Conclusion:**

Further improvements in water fitting technology could possibly solve the problem of bacterial water contamination in health care settings.

## Background

The use of water in health-care facilities may lead to increased risk of healthcare associated infection through bacterial proliferation in water piping and water fittings, and is particularly documented in vulnerable population groups such as the young, the elderly and immunocompromised patients [1,2]. The World Health Organization had placed particular emphasis on water safety in health care facilities [3] because of the concentration of susceptible persons in these, as compared with, domestic settings. Amongst other microorganisms, *Pseudomonas aeruginosa* has been reported as having a significant health impact in hospitals and nursing homes, resulting in longer hospital stays and deaths [4]. Drinking water that is used for cleaning machines, wound cleaning, and other procedures that bring water in contact with patients provides a potential transmission route for these microorganisms.

In the past decade, particularly sensor-operated non-touch fittings have been increasingly used also in hospitals, chiefly because of two aspects: from an infection control point to prevent or reduce contamination of hands after hand washing, and from an economic point to reduce up to 40% of water consumption by shortening the time of water flow. Regretfully, the latter aspect together with the technical requirement that waterlines and valves of most non-touch water fittings are made of plastic materials may promote a rapid bacterial accumulation [5], which is extremely difficult to eradicate once it has been established. Water fittings with brass valve blocks have not been studied so far.

Aside from the materials used, easily modifiable factors that may influence the prevalence and extent of the microbial contamination and accumulation of non-touch-fitting-systems are the frequency of usage and the duration of water flow during and after hand washing. Because of this, water-fitting manufacturers have started to provide programmable non-touch fittings that allow regular automated post-flushing with cold water to prevent water stagnation. However, the ideal duration of post-flushing is unknown. Therefore, the aim of this experimental laboratory based study was to investigate the frequency of usage, the duration of water stagnation, and various post-flushing times after use of non-touch fittings which incorporate brass valve blocks instead of plastic material, on the total bacterial count/mL water. Additionally, the effect of pre-rinsing with cold water before use was explored.

## Methods

### Non-touch water fitting

The non-touch water-fitting model Manutronic (Dallmer Ltd, Lavenham, UK) was used for the experiments. The fitting is equipped with a Differential Dynamic Signal Absorption (DDSA) technology (Oblamatik GmbH, Switzerland), which is based on a sensor field surrounding the fitting and tracks the user’s hand even without him having to be aware of a sensor trigger mechanism. Hence, the Manutronic fitting itself serves as a sensor and adapts itself quickly to a user approaching from any direction. One of the key-features of the Manutronic non-touch fitting is its ability for customising the fitting operation for triggering on approach, non-contact temperature adjustment and a cold rinse feature at various time interval settings. There are no visible controls in or on the fitting outlet, thus cleansing or disinfecting cannot damage the sensor. Finally, the fitting uses a brass valve block with no plastic material.

### Experimental setting

**Figure 1:**
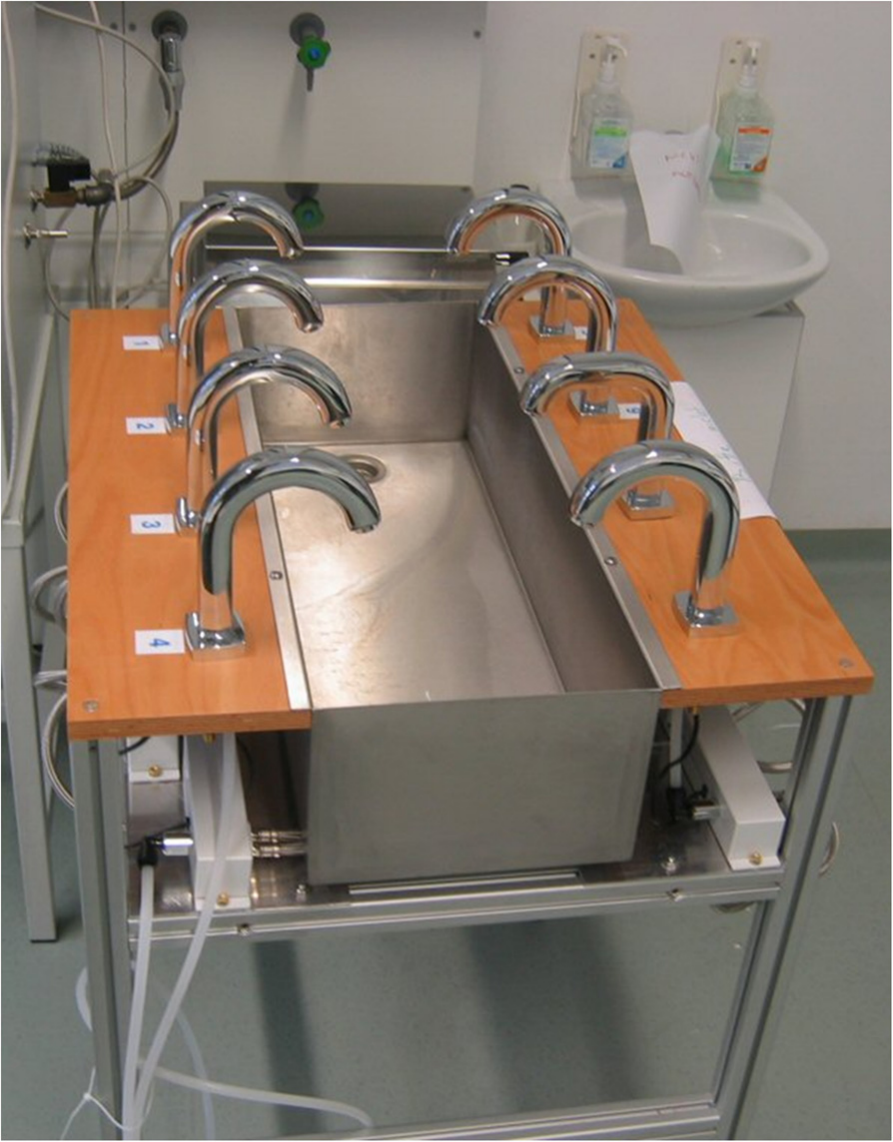
Laboratory set-up of non-touch sensor fittings on a mobile water sink.

Eight non-touch fittings were mounted on a mobile test sink and connected to the same central water pipe source (Figure 1). The central water pipe was connected to the municipal drinking water supply of the City of Vienna, Austria. The set-up of the 8 fittings differed only in presence or absence of water connection pipes, length of connection pipe, frequency of usage, and time intervals for pre- and post-usage water flush (Table 1). The set-up of fittings no. 1 and 2 were used to test the potential influence of a frequency of usage for 30 seconds, once weekly or once monthly, respectively. Fittings no. 3 and 4 served to examine the potential influence of daily usage (10 times use for 30 seconds, each) and the influence of the length of stagnating water in the connection pipe. Fittings no. 5 and 6 allowed investigation of a potential effect of rinsing the fitting by programing an automatic post-flushing with cold water at 18°C for 2 or 10 seconds, respectively. And finally, fitting no. 7 allowed assessment of a possible influence of the used valve block material, and fitting no. 8 served to evaluate the possible effect of rinsing the fitting by programming an automatic pre-flushing with cold water for 30 seconds before use. This fitting also served as a “control”, representing bacterial accumulation (total cfu count/mL) occurring at a water fitting site with low frequency usage. The primary question at study was to investigate possible differences in bacterial counts obtained from regularly used fittings (fittings no. 3–7) with particular respect to no post-flushing and 2 seconds or 10 seconds of automated post-flushing with cold water.

**Table 1:** Details of tested variation in presence or absence of connection water pipe, frequency of use, and time interval of pre- and post-usage water flush

**Fitting no.**	**Connection pipe length**	**Frequency of usage **^**a**^	**Mode of flushing**	
			**Pre-flushing**	**Post-flushing **^**b**^
1	50 cm	once weekly	none	none
2	50 cm	once monthly	none	none
3	50 cm	10× daily	none	none
4	300 cm	10× daily	none	none
5	50 cm	10× daily	none	2 s
6	50 cm	10× daily	none	10 s
7	none	10× daily	none	none
8	50 cm	none	30 s	none

^a^ usage: 30 seconds at a pre-set water temperature of 37°C.

^b^ cold water at a pre-set water temperature of 18°C.

### Water collection and microbiological analysis

Cold water samples (500 mL each) were collected once weekly over a period of 20 weeks from fittings no. 1 and no. 3 to 8, and once monthly from fitting no. 2. Sampling was performed randomly, and not by the sequential number of the water fittings. Water was collected directly into sterile flasks from the fittings without any manipulation or pre-rinsing (except for fitting no. 8) and without removing or disinfecting the aerator. This measure allowed to assess the microbiological water quality as it would have come in contact with a hand under the respective fitting.

The total bacteria colony-forming unit (cfu) counts were obtained by the spread plate technique. One mL of each water sample was directly inoculated on a Caseinpeptone soyapeptone agar (CSA; Merck, Germany). For detection of *Pseudomonas aeruginosa*, 100 mL of each water sample was filtered through a cellulose-nitrate filter (Sartorius, Germany; pore size: 0.45 μm). The cellulose-nitrate filters were placed on a CSA. All samples were incubated at 36 ± 1°C for 48 hours and at 22 ± 1°C for further 4 days. After a total incubation time of 6 days, cfu were counted per 1 or 100 mL of water sample, respectively, and denoted as cfu/mL.

### Statistical analysis

The geometric mean / scatter factor over time was calculated for all fittings. For this purpose and to obtain conservative estimates, 0 cfu/mL results were replaced by 0.5 and counts above 1,000 by 1,000. Statistical analyses were performed using SPSS software, version 18.0 (IBM Corp, NY, USA). For fittings no. 3–7, as those were the fittings used 10 times daily, analysis of variance was performed on log-transformed cfu/mL. Normality of residuals was tested by Kolmogorov-Smirnov test with Lilliefors corrected p-values. Homogeneity of variances was tested by Levene’s test. Post hoc test of differences between fittings was done by Tukey’s honest significant difference tests. A p-value of less than 0.05 was considered to be statistically significant.

## Results

The detailed numbers of cfu/mL, stratified by tested fitting, are summarized in Table 2. Only fittings no. 6 and no. 8 showed no total cfu/ml counts higher than 100 cfu/mL, which is the microbiological threshold for water of drinking quality [6]. Moreover, fitting no. 8 did not surpass the restrictive threshold of 20 cfu/mL at 37°C. Hence, the microbiologically best setting to prevent a non-touch fitting colonization was a short water connection pipe and a regular pre-flush of 30 s before water sampling, even if the fitting was not used regularly for hand washing.

**Table 2:** Number of cfu/mL retrieved from fittings no. 1-8

**Weeks**	**no. 1**	**no. 2**	**no. 3**	**no. 4**	**no. 5**	**no. 6**	**no. 7**	**no. 8**
0	0	0	0	0	0	0	0	0
1	0	-	0	0	0	0	0	-
2	> 1,000	-	0	2	67	15	0	-
3	> 1,000	-	0	0	0	0	124	-
4	> 1,000	> 1,000	0	0	0	0	0	0
5	> 1,000	-	150	>1,000	46	0	100	-
6	> 1,000	-	62	400	123	14	300	-
7	> 1,000	-	1	1	10	1	10	-
8	> 1,000	> 1,000	162	200	152	22	500	-
9	> 1,000	-	69	226	30	0	167	4
10	> 1,000	-	4	17	1	1	170	0
11	> 1,000	-	317	67	147	6	286	5
12	> 1,000	> 1,000	17	21	14	0	252	0
13	> 1,000	-	89	139	30	0	192	1
14	> 1,000	-	87	46	402	1	90	0
15	> 1,000	-	95	15	10	16	226	0
16	> 1,000	> 1,000	20	1	1	1	55	1
17	> 1,000	-	200	259	321	11	500	3
18	> 1,000	-	180	117	237	26	118	2
19	> 1,000	-	290	109	150	40	250	0
20	> 1,000	-	17	0	0	0	0	-
Geometric mean	-	-	17.8	15.4	14.2	2.2	39.7	1.0
Scatter factor	-	-	11.9	14.6	12.2	5.4	14.0	2.4

Comparing fittings that were used 10 times per day, there were no statistically significant differences between fittings except for fitting no. 6, which showed significantly lower cfu/mL counts compared to all other fittings (Table 3). Fitting no. 6 differed from the others by a longer post-flushing of 10 s. *P. aeruginosa* results were < 1 per 100 mL for all samples.

**Table 3:** Tukey HSD-test of log10 cfu between fittings no. 3-7

**Fitting**	**3**	**4**	**5**	**6**	**7**
3		1.0000	0.9997	0.0006	0.1900
4	1.0000		0.9995	0.0006	0.1988
5	0.9997	0.9995		0.0011	0.1271
6	0.0006	0.0006	0.0011		0.0001
7	0.1900	0.1988	0.1271	0.0001	

## Discussion

Outbreaks of healthcare associated infection due to tap-water contamination have been frequently reported. An outbreak of 14 cases of urinary tract infections caused by *Pseudomonas aeruginosa* occurred in a paediatric surgical department [7]. Multiple *P. aeruginosa* isolates were also found in the tap water, as the only putative source for infection in patients. Molecular typing indicated a possible direct contamination of patients via the distal colonization of fittings with *P. aeruginosa*.

The presence of outbreak strains in tap water, not including only *P. aeruginosa* but also other microorganisms, seem to be an important factor in subsequent dispersion and potential disease transmission within a healthcare setting [8]. Regular disinfection of contaminated water lines, water fittings, and change of water aerators in critical areas can reduce the number of colony-forming units and was shown to reduce the number of healthcare associated infections due to water-borne pathogens [9]. An effect on the total plate count may also affect *P. aeruginosa* counts, therefore emphasis was laid on the total cfu counts/mL water.

However, when such measures have been successful, eradication was achieved only after excessive procedures and at high costs [5,9,10]. Therefore, instead of secondary control or tertiary outbreak management measures, primary prevention of bacterial colonisation and excessive accumulation may be more appropriate. To prospectively prevent bacterial accumulation in water, it is important to understand factors leading to it and to avoid such factors during the phase of construction [11].

Several factors have been identified resulting in bacterial colonisation and accumulation in water fixtures. In 2001, Halabi et al. [12] reported the association of non-touch fittings in hospitals and water contamination with *P. aeruginosa* and *Legionella spp*.. The authors studied the bacteriological water quality of 2 different non-touch water fittings types, 23 fittings without temperature selection and 15 with temperature selection (cold and warm). Seventy four percent of the taps without temperature selection and 7% of the taps with temperature selection showed contamination with *P. aeruginosa* (P < 0.001). The authors found the source of contamination to be the magnetic valve made of plastic materials and the fitting outlet itself, whereas the junction from the central pipe system was free of contamination. It was concluded that material selection together with low amount of water flow through the outlets, a low water pressure and the length of water stagnation at a temperature of 35°C were responsible for the excessive development of biofilms in non-touch fittings. Lepart et al. [13] later confirmed these findings that non-touch fittings without temperature selection provide ideal growth conditions for *P. aeruginosa*. Indeed, in our study water fittings with brass valve blocks were used. This may possibly be one explanation why we did not yield any *P. aeruginosa* in our samples. Further research is needed to compare fittings with plastic or brass valve blocks.

To investigate if bacterial accumulation in water fittings is a particular problem associated with non-touch fittings, Assadian et al. [14] conducted a study comparing 4 different non-touch fitting models against standard handle-operated fittings. This case-controlled study could not confirm a statistically significant difference in *P. aeruginosa* counts between non-touch and handle-operated fittings. *P aeruginosa* was retrieved only from a standard fitting in a neonatal intensive care unit with irregular use and reduced water flow.

Therefore, it remained debateable whether bacterial colonization and accumulation are supported by a flushing effect, the frequency of use, or the length of a stagnating, tempered water column. Because of this the aim of the present experimental study was to examine the influence of the frequency of usage, the duration of water stagnation, the influence of brass valve blocks, and post or pre-rinsing with cold water before or after use of non-touch fittings in a controlled laboratory setting.

## Conclusions

The results of this study demonstrate that a negative effect was associated with a decreased frequency of use in combination with a long stagnating water column. Cold water post-flushing for 10 seconds, however, effectively was able to prevent non-touch fittings from bacterial accumulation over a period of 20 weeks. The effective time necessary to achieve such preventive effect may range somewhere between 2 and 10 seconds. Pre-flushing of 30 seconds with cold water was the most effective method to prevent bacterial accumulation in the tested non-touch fittings. It seems however impossible to implement this measure into a busy clinical real-life setting. The water fitting would have to “know” about 1 minute before that somebody needs to wash hands. Such precognition is not achievable, and certainly technically not achievable by today’s technology.

Furthermore, flushing with cold water, either pre- or post-usage of a fitting, is only realizable with electronically operated fittings, since the required consistency is not expectable from human users. Such a concept would have further advantages: since most relevant pathogens causing healthcare associated infection are mesophilic with growth optimum ranging between 20°C and 45°C, an automated flushing program with a cold water flush for 5–10 seconds, which is triggered when the water temperature in a stagnating water column to the fitting increases above e.g. 15°C, could possibly solve the problem of bacterial water contamination and accumulation in health care settings in future.

## Competing interests

The authors declare that they have no competing interests.

## Authors’ contributions

MS and OA formulated the study questions. MS, OA, and MK participated in designing the study. MK performed the statistical analysis. MS performed all experimental tests. MDE participated in analysis of results and manuscript preparation. All authors read and approved the final manuscript.

## Pre-publication history

The pre-publication history for this paper can be accessed here:

http://www.biomedcentral.com/1471-2334/13/402/prepub
